# “Stay at Home” during the COVID-19 Pandemic: Effects on Physical Activity and Sedentary Behavior in an Italian Academic Community

**DOI:** 10.3390/ijerph20021168

**Published:** 2023-01-09

**Authors:** Anna Mulasso, Margherita Micheletti Cremasco, Simone Cuomo, Andrea Testa, Lynda Stella Lattke, Aurelia De Lorenzo, Alberto Rainoldi, Emanuela Rabaglietti

**Affiliations:** 1 NeuroMuscular Function Research Group, Department of Medical Sciences, School of Exercise and Sport Sciences, University of Torino, 10126 Torino, Italy; 2 Department of Life Science and Systems Biology, University of Torino, 10123 Torino, Italy; 3 School of Exercise and Sport Science, University of Torino, 10126 Torino, Italy; 4 Department of Psychology, University of Torino, 10124 Torino, Italy

**Keywords:** pandemic, COVID-19, active lifestyle, physical activity, sedentary behavior, adult population, Italian context

## Abstract

This study investigates changes in physical activity (PA) and sedentary behavior in an Italian university community during Phase 1 of SARS-CoV-2 lockdown (“stay at home” government decree, from March 8th to May 4th, 2020) compared to their habits prior to the COVID-19 pandemic. We also examine differences according to gender, university position, BMI categories, and sport participation. A total of 2596 people (median age 24, IQR 11 years; 70.8% women) filled out a survey after eight weeks of statutory confinement at home. The International Physical Activity Questionnaire measured PA and sedentary behavior in a typical week before and during lockdown. Physically inactive people passed from 10.9% to 35.0% before–during the lockdown. The total amount of PA decreased (median 2307 vs. 1367 MET-min/week; *p* < 0.001), while sedentary behavior increased (median 349 vs. 440 min/day; *p* < 0.001) between before and during the lockdown. The following categories showed a higher reduction in the total amount of PA: men when compared to women; people with normal weight when compared to pre-obese/obese people; and people who played sports when compared to those who did not play sports. There is a need to propose PA/exercise programs to counteract physical inactivity and sedentarism during a social emergency, with special attention to people who showed higher PA reduction.

## 1. Introduction

By the end of December 2019, a new coronavirus called SARS-CoV-2 from Hubei Province (Central China) began to spread rapidly and unexpectedly to humans, becoming a serious threat to the health of the entire world. On 11 March 2020, the World Health Organization (WHO) declared the start of the SARS-CoV-2 pandemic [1]. Since then, and as of the 1st half of October 2022, there have been more than 618 million confirmed cases and 6.5 million deaths worldwide [2]. Thus far, according to WHO (2022), Europe is the most affected continent with over 250 million infected people and, after the Americas, it records the second highest number of victims (over 2,100,000 on 14 October 2022) [3].

In Italy, the first two patients diagnosed with SARS-CoV-2 were identified on 30 January 2020 [4]. The situation gradually worsened until it finally exploded between March and April of that same year. Italy was the eighth country by the number of infections and the fifth by the number of victims of the entire world [3]. By 8 March 2020, the Italian government imposed strict measures to contain the spread of the virus in the worst-affected areas (mainly the regions of Northern and Central Italy) [5], and, the day after, these measures were extended throughout the whole country [6]. In this period of lockdown (which was known as Phase 1 in Italy), the government imposed a “stay at home” order, limiting mobility outside the home to what was deemed as a basic need (i.e., food shopping, medical care, and essential jobs). Most businesses were temporarily closed yet many, depending on the type of service, continued their work virtually through smart technologies. Any form of assembly in public and private environments was forbidden. Sport centers, gyms, and public parks were closed. These restrictions remained in effect for eight weeks until 4 May 2020. After this date, began Phase 2. It was characterized by the gradual reopening of services and, above all, by the mobility of citizens and the possibility of being outdoors with fewer restrictions [7]. Although these measures were effective in containing viral infections [8,9,10,11,12], the imposition of such a strict lockdown has negatively affected people’s well-being and health (i.e., people changed their behavior in terms of physical activity, eating habits, sunlight exposure, socialization, mood, etc.) [13,14,15,16,17,18,19,20,21,22,23]. In this context, Mattioli et al. (2020) noted that during the lockdown, “loss of freedom, uncertainty over disease status, and boredom can create dramatic effects” [17] (p. 852).

To counteract these negative effects, the WHO recommended remaining physically active during the lockdown (i.e., at least 150 min per week of moderate physical activity, or 75 min of high intensity activities). Examples include online exercise, aerobic training exercise, jumping rope, dancing, active video gaming, “muscle and balance” exercises [24]. According to WHO, physical inactivity is the fourth risk factor for mortality after hypertension, tobacco use, and high blood glucose levels [25]. Those who do not reach the recommended physical activity (PA) level, have a higher risk (+20–30%) of cancer, cardiovascular diseases, stroke and diabetes, and a potential loss of 3–5 years of life [25].

Even under “normal” circumstances, levels of PA are below recommended health threshold in about 25% of adults worldwide [26,27]. Similarly, Owen et al. (2010) reported that one in four USA adults spends about 70% of their time in sitting position [28]. In Italy, levels of PA are inadequate for 41.4% of adults (36.2% for men and 46.2% for women) [29]. During the lockdown, it seems that people spent more time in sedentary activities at the expense of habitual amounts of PA [18,30,31,32]. Pecanha et al. (2020) reported a reduction in average step count detected using the FitBit, Inc. (San Francisco, CA, USA) from 38% to 7% when comparing the same week of March 2020 to March 2019 in more than 30 million users all over the globe [30]. Additionally, Tison et al. (2020) found an average decrease in the number of daily steps of 5.5% and 27.3% after 10 and 30 days of lockdown, respectively [33]. A consistent number of studies (among these: Bowes et al., 2020; Cancello et al., 2020; McCarthy et al., 2021; Stockwell et al., 2021; Wang et al., 2020) reported that more than 50% of people examined were less active during the lockdown in comparison to previous periods [34,35,36,37,38].

Studies on the Italian population related to “Phase 1” of SARS-CoV-2 pandemic are still limited [39,40,41,42,43,44,45,46,47,48]. Overall, they confirm a general reduction in PA [39,40,41,42,43,44,45,47,49], while a limited amount of evidence reported an increase in recreational PA [42], a high percentage of people (22.7%) who started training during the lockdown [46], and an increase in training frequency during the lockdown in people who already played sports [48]. In particular, all these studies have involved undergraduates [44,45], University samples of both students and employees (University of Florence and the University of Naples Federico II, respectively) [42,43], patients affected by neuromuscular disease [40], and heterogeneous Italian populations [39,40,41,46,47,48,49]. Most of the data were collected a few weeks after the beginning of Phase 1 [41,44,45,46,47,48,49], using single questions (not validated measures) to quantify physical activity [39,43,46,48]. A limited number of studies considered the time spent in sedentary activities [42,43,44,45,49]. Given the mixed survey periods and assessment methods, more studies are required to understand and generalize the impact of the COVID-19 pandemic on PA and sedentary behavior in the Italian population. Consequently, this article focuses on a well-defined community that includes university students and employees with the same socio-cultural work environment. Since our survey was completed after two months of lockdown (i.e., immediately after “Phase 1” had begun), the data collected reflects all the negative consequences of “Phase 1”. Furthermore, a validated questionnaire was used to detect quantity of PA and sedentary behavior.

Therefore, this article aims to investigate changes in the quantity of PA and level of sedentary behavior in an Italian university community during “Phase 1” of the SARS-CoV-2 lockdown compared to their habits prior to the pandemic. All the university members (e.g., students, post-graduate, academic, and administrative and technical staff) were invited to participate. The following two specific aims were achieved: (i) analyze differences in all subtypes of PA-total, vigorous, moderate, and walking, and changes in levels of sedentary behavior in the periods before and during the lockdown; (ii) examine these same differences in the subtypes of PA and sedentary behavior according to individual characteristics, such as gender, university role, BMI categories and participation in sports.

## 2. Materials and Methods

### 2.1. Study Population and Procedures

This large-scale cross-sectional study was conducted at the University of Torino (UniTO; https://www.unito.it) (accessed on 7 September 2020), which is located in a walkable city with several green spaces. UniTO has about 70,000 students, 4000 academic, administrative and technical staff, and 1800 post-graduate and post-doctoral students. The entire university community received an email introducing the project and inviting them to participate. Participants included students (e.g., undergraduate students, Master’s degree students, etc.) and employees (e.g., technical and administrative staff, researchers, professors, etc.). The online survey was conducted from 14 May to 31 May 2020, immediately after 8 weeks—from 8 March to 4 May 2020—of statutory confinement at home as decreed by the Italian government (called “Phase 1” and translated as “#stayathome decree”) [6]. The survey was written in Italian and conducted through the Google Forms platform. Before filling out the questionnaire, participants had to read and accept the consent form. All data were collected with anonymity. The Ethics Committee of the University of Torino approved the study protocol (Protocol code 179496). No rewards or incentives were offered for participating.

### 2.2. Measures

The International Physical Activity Questionnaire (IPAQ, 7-items) was used to measure PA [50,51]. The items of the IPAQ assess the time spent in the following activities in a typical week before and during the SARS-CoV-2 lockdown: (i) vigorous physical activities, such as heavy lifting, digging, and aerobics, (ii) moderate physical activities, such as carrying light loads, cycling at a steady pace, (iii) walking (for at least 10 min at a time), and (iv) sitting. PA was reported as both continuous (METs, metabolic equivalents of oxygen consumption, per min/week) and categorical (physically inactive, active, highly active) values, calculated as specified in the IPAQ (2005) Data Processing and Analysis Guidelines [52].

In addition, sociodemographic characteristics (e.g., age, gender, education level, university role, etc.), anthropometric variables (e.g., height, weight), information about the health status (i.e., SARS-CoV-2 infection) and sports participation in the month before the epidemic were collected.

### 2.3. Statistical Analysis

Statistical analyses were performed using the Statistical Package for Social Sciences (SPSS), version 26.0 (SPSS Inc., Chicago, IL, USA). The level of statistical significance was set at alpha <0.05. Descriptive statistics were obtained for all variables in the study.

As suggested in the Guidelines for Data Processing and Analysis of the IPAQ (2005) [52], and considering the non-normal distribution of our data, continuous values of IPAQ were presented as median and quartiles (Q1–Q3), expressed in MET-minutes/week. The IPAQ question on sitting time was also presented as median and quartiles in minutes. The standard method of data cleaning was adopted, i.e., responses such as “Do not know/Not sure” were not used in the analysis. A sample of N = 2596 successfully answered the IPAQ questions on vigorous, moderate, and walking physical activities, of which *n* = 2275 also reported the time spent sitting.

The Stuart–Maxwell test was performed to compare non-dichotomous categorical IPAQ scores (Physically inactive, Active and Highly active) in the period before and during the lockdown [53,54]. In addition, Wilcoxon’s signed-rank tests were performed to identify any differences in the period before and during the lockdown for different subtypes of PA-total IPAQ, vigorous PA, moderate PA, walking activities (expressed as MET-minutes/week), and sedentary behavior (expressed as minutes/day). Mann–Whitney U tests for two independent groups or Kruskal–Wallis H tests for three independent groups were performed to examine whether the changes, Δ (operationalized as the difference between before and during the lockdown), for the total amount of PA, vigorous PA, moderate PA, walking activities, and sedentary behaviors differed based on university role (employees or students), gender (female or male), BMI categories (underweight, normal-weight or pre-obese/obese), and sports participation (Yes or No). For each statistically significant difference, the effect size (ES) was calculated to assess sensitivity to change. For Wilcoxon’s signed-rank tests and Mann–Whitney U tests, a non-parametric effect size, calculated as *r* = |*z*|/*Vn*, was applied [55]. Values of r below 0.3 were considered small, 0.3–0.5 medium, and higher than 0.5 large [56,57]. For the Kruskal–Wallis H test, the eta-squared measure (*η*^2^ = *H* − *k* + 1/*n* − *k*) was calculated [55]. Values of *η*^2^ were interpreted as small (0.01–<0.06), moderate (0.06–<0.14) and large (≥0.14) effect [58,59,60].

## 3. Results

### 3.1. Baseline Characteristics of Participants

Table 1 shows the characteristics of the study participants (*N* = 2596). The total sample had a mean age of 30 years (SD = 12; median of 24 years, IQR 11 years). Approximately two-thirds of participants were women (*n* = 1837, 70.8%) and university students (*n* = 1975, 76.1%). Sixty-seven percent (*n* = 1334) of students had an educational level equivalent to a High school diploma, while 51.9% (*n* = 322) of employees had a university degree. A high percentage of the sample was of normal-weight (73.4%) and played sports in the month before the epidemic (70.8%). A limited number of participants (*n* = 44; 1.7%) were infected with the SARS-CoV-2 virus.

### 3.2. Differences in PA and Sedentary Behavior before and during the Lockdown

Considering the level of PA, 283 (10.9%) individuals were classified as Physically inactive, 1261 (48.6%) as Active, and 1052 (40.5%) as Highly active during the period before the lockdown. Overall, the lockdown period resulted in a reduction in the PA level for 40.0% (*n* = 1039) of the sample (data reported below the diagonal–Table 2), while 48.8% (*n* = 1266) showed no change (data reported on the diagonal–Table 2) and 11.2% (*n* = 291) increased their PA level (data reported above the diagonal–Table 2). Specifically, 65.7% (*n* = 186) of the Physically inactive, 44.3% (*n* = 559) of the Active and 49.5% (*n* = 521) of the Highly active maintained the same PA level as before the lockdown. Among these, 910 (35.0%) subjects were classified as Physically inactive, 931 (35.9%) as Active, and 755 (29.1%) as Highly active during the lockdown. For the entire sample, the Stuart–Maxwell test determined that categorical IPAQ scores were significantly different before and during the lockdown (*p* < 0.001).

When analyzing the entire sample, the Wilcoxon’s signed-rank tests showed significant differences in the total amount of PA between before and during the lockdown (median 2307 vs. 1367 MET-min/week; *p* < 0.001; ES = 0.50). Specifically, vigorous PA (median 960 vs. 480 MET-min/week; *p* < 0.001; ES = 0.20) and walking activities (median 693 vs. 99 MET-min/week; *p* < 0.001; ES = 0.69) performed during the lockdown were lower compared to the previous period. No differences were observed for moderate PA (median 240 MET-min/week for both periods). Sedentary behavior increased significantly (median 349 vs. 440 min/day; *p* < 0.001; ES = 0.57) between before and during the lockdown. Table 3.

The total amount of PA showed a statistically significant reduction between before and during the lockdown in all subgroups of participants, with ES values ranging from 0.22 (individuals who did not participate in sports; median 1282 vs. 840 MET-min/week; *p* < 0.001) to 0.60 (male gender; 2895 vs. 1600 MET-min/week; *p* < 0.001). Analyses between groups revealed differences in changes in total PA based on gender, BMI, and exercise participation. Indeed, men (median reduction 933 (0–2310) MET-min/week) showed higher changes than women (median reduction 643 (−80–1732) MET-min/week; *p* < 0.001); individuals with normal-weight (median reduction 759 (0–1983) MET-min/week) compared to pre-obese/obese individuals (median reduction 632 (−130–1642) MET-min/week; *p* = 0.013); individuals who exercised (median reduction 977 (0–2280) MET-min/week) compared to individuals who did not exercise (median reduction 185 (−278–1004) MET-min/week; *p* < 0.001).

A significant decrease was also observed for vigorous PA in all subgroups before and during the lockdown, except for those who did not exercise (on the contrary, they increased the amount of vigorous PA; *p* < 0.001 with ES = 0.29). ES values ranged from 0.15 (underweight category; 480 vs. 240 MET-min/week; *p* < 0.001) to 0.35 (male gender; 1440 vs. 800 MET-min/week; *p* < 0.001). Gender and sport participation showed significant changes in the vigorous PA between groups. Men (median change 0 (0–1280) MET-min/week) had a greater decrease compared to women (median change 0 (−240–640) MET-min/week; *p* < 0.001), and individuals who exercised (median reduction 160 (0–1200) MET-min/week) compared to those who did not (median change 0 (−480–0) MET-min/week; *p* < 0.001).

Considering the moderate PA, only a limited number of subgroups show significant differences between before and during the lockdown. Specifically, men and individuals who exercised decreased the amount of moderate PA (EF of 0.16 and 0.14, respectively; both with *p* < 0.001); whereas individuals who did not exercise increased the amount of moderate PA (0 vs. 160 MET-min/week; ES = 0.28; *p* < 0.001). As for vigorous PA, gender and sport participation also showed significant changes between groups for the moderate PA. Men showed a greater reduction (median change 0 (−120–280) MET-min/week) compared to women (median change 0 (−240–200) MET-min/week; *p* < 0.001) and individuals who exercised (median change 0 (−160–280) MET-min/week) compared to those who did not (median change 0 (−320–0) MET-min/week; *p* < 0.001).

Regarding walking activities, all subgroups reported a significant decrease in this quantity between before and during the lockdown. ES values ranged from 0.63 (pre-obese/obese subjects; median 693 vs. 99 MET-min/week; *p* < 0.001) to 0.71 (underweight subjects; median 792 vs. 99 MET-min/week; *p* < 0.001). Gender and BMI categories showed significant changes in walking activities between groups. Females had a greater decrease (median reduction 495 (99–1040) MET-min/week) compared to males (median reduction 429 (0–924) MET-min/week; *p* < 0.05), and individuals with normal-weight (median reduction 495 (99–1040) MET-min/week) compared to individuals with pre-obesity/obesity (median reduction 396 (0–924) MET-min/week; *p* < 0.05).

Finally, sedentary behavior changed negatively before and during the lockdown. In all subgroups, time spent in sedentary activities increased significantly, with ES values ranging from 0.50 (individuals who did not exercise: median 360 vs. 440 min/day; *p* > 0.001) to 0.63 (male gender; median 300 vs. 440 min/day; *p* > 0.001). When analyzing differences between groups, only individuals who exercised showed a greater increase in sedentary behavior (median reduction −80 (−180–0) min/day) compared to individuals who did not exercise (median reduction −60 (−180–0) min/day; *p* ≤ 0.05). Table 3.

## 4. Discussion

This paper explored the effects of the lockdown (“Phase 1” of SARS-CoV-2, from 8 March 2020 to 4 May 2020) on PA–total, vigorous, moderate, and walking activities, and sedentary behavior in an Italian university, comparing data from the period before and during the lockdown. Differences in PA and sedentary behavior across different subgroups of participants (i.e., male/female, students/employees, underweight/normal-weight/pre-obese, and obese individuals who did/did not exercise) were also examined.

Although the WHO and other national/international scientific organizations recommended staying active during the lockdown, our study showed that 40.0% of the participants reduced their level of PA (40.3% of the Active became Physically inactive, and 20.5% and 29.9% of the Highly active switched to Physically inactive and Active levels, respectively). Before the lockdown, the proportion of Inactive individuals was 10.9%. During the lockdown, it increased to 35.0%. Consistent with other studies summarized in the review by Stockwell et al. (2021) [37], our participants decreased the total amount of PA, particularly the vigorous and the walking activities; on the contrary, they spent more time in sedentary activities. This is not surprising because with the lockdown, unnecessary outdoor activities were prohibited, and many adults worked from home. Students attended online lessons from home, resulting in a reduction in movement (e.g., commuting from home to work) [61], an increase in total screen time [62] and sedentary behavior [44,63]. In addition, gyms and all sports facilities were closed, limiting the opportunity to perform any PA, specifically vigorous PA. Instead, no significant changes in moderate physical activities were found in the total sample, as has been the case in other studies (including Castañeda-Babarro et al., 2020; and Romero Blanco et al., 2020) [64,65]. This result can likely be attributed to the fact that alternative activities, such as housework, gardening, etc., that require moderate effort increased during the lockdown, keeping the proportion of moderate PA unchanged during the lockdown.

Regarding university participants’ role, students and employees decreased the total amount of PA, vigorous, and walking activities, and increased sedentary behavior between before and during the lockdown, showing the same trajectory related to lifestyle habits. In this regard, our findings are only partially consistent with previous studies (including a university community of students and employees) [66]. For example, Barkley et al. (2020) found an increase in sedentary behavior, but a decrease in PA exclusively among students/employees who were most active [66]; and De la Rosa et al. (2022) showed that students decreased the amount of PA more than employees [67]. A direct comparison of these results is difficult because of differences in methodology, sampling and type of lockdown across countries (i.e., Barkley et al. in the Midwestern United States, and De la Rosa et al. in Colombia).

Instead, both men and women decreased the total PA, the vigorous PA and the walking activities, suggesting an increase in sedentary behavior between before and during the lockdown. In contrast, only men decreased the moderate PA during the lockdown. It should be noted that women and men showed differences in the trajectories of change in the total amount of PA, vigorous PA, moderate PA, and walking activities. In all these cases, consistently with previous studies [41,49], men reported a greater PA reduction than women. Since gender differences in performing PA have been extensively discussed in the literature [68,69], our results may be explained by the fact that men are more inclined to engage in competitive and outdoor activities [69], which they sought to avoid during the lockdown. Furthermore, men are more active in their leisure time than women [68], and are more likely to engage in vigorous PA [68]. In contrast, women prefer doing PA at home [41] and are more engaged in housework than men [68]. Women can counteract the reduction in PA during the lockdown by engaging in these activities.

Among individuals with different BMI, all three categories (underweight, normal-weight, and pre-obese/obese) decreased the total amount of total PA, vigorous PA, and walking activities, and increased the time spent in sedentary activity, as reported by previous authors [49,70]. Exclusively, normal-weight individuals also decreased moderate PA during the lockdown. When analyzing the pathways of change in the three subgroups, we found that normal-weight individuals significantly reduced total PA more than pre-obese/obese individuals. This may be explained by the fact that individuals who were more active in the period before the lockdown, as reported in the normal-weight category, were more likely to report a more extensive decrease in PA [37].

As expected and consistent with other studies [36,37], we also found that individuals who played sports had a greater decrease in total, vigorous and moderate PA, and a greater increase in sedentary behavior than subjects who did not exercise. In general, both subgroups (individuals who played sports/did not play sports) showed a decrease in total PA, and vigorous, moderate, and walking activities during the lockdown, whereas the time spent in sedentary behavior increased for both.

The present findings should be interpreted in the context of some limitations. First, data on PA and sedentary behavior were obtained from a self-report questionnaire. Consequently, it is questionable whether participants were able to objectively assess and correctly recall previous activities, especially those performed before the lockdown. On the other side, it was not possible to collect device-based measures that required face-to-face contact during the strict lockdown imposed by the national government. Second, the sample consisted mainly of women. This is likely because women have a different level of attention and concern for lifestyle and health issues than men, as suggested in other similar studies [44,71]. Furthermore, the response rate could have been higher. The low response rate makes it impossible to generalize the results to the entire university community and/or, even less, to consider them representative of the general Italian population of young adults and adults. Lastly, the survey focused mainly on individual factors that may influence PA and sedentary behavior, and did not examine other aspects (i.e., social, environmental, etc.) that may also influence people’s lifestyles and could have provided a more comprehensive picture of the phenomenon.

The fact that the survey was conducted immediately at the end of the “Phase 1” (which lasted two months) of the SARS-CoV-2 pandemic was a strength of the study. In Italy, Phase 1 represented the most critical phase of the pandemic, characterized by the imposition of a strict lockdown (which was not followed by other such strict national lockdowns). The opportunity to survey the consequences of the entire period was a unique and unrepeatable opportunity. In addition, the survey used validated measures, it was easily accessible to the whole university community, and it required a minimum amount of time to fill out.

From a practical point of view, the findings of our study highlight the urgent need for preventive strategies suitable for increasing the quantity of PA and to reducing sedentary behavior during periods when people are confined to their homes (e.g., smart working, illnesses, etc.). As previous studies have shown, low levels of PA and high levels of time spent in sedentary behavior negatively affects people’s health (both physically and psychologically) and well-being [72,73]. To counteract physical inactivity and sedentary behavior, simple PA/exercise programs can be easily proposed, also making use of apps/web-based interventions as a training guide. For instance, it is possible to perform aerobic and resistance training in association with balance, range of motion, and flexibility exercises using materials available at home (e.g., sticks, bottles, rubber bands, etc.), by varying the stimuli according to the target group. Special attention should be paid to individuals who were physically inactive and to those who had a greater reduction in PA, such as men and/or individuals who exercised. In such cases, it would be more appropriate to propose a personalized and targeted PA/exercise program that can also act on motivation. Community-based lifestyle strategies and policies to support PA and reduce sedentary behavior should also be encouraged. Initiatives can be heterogeneous, multidimensional, and specific to target groups that have different behavioral routines (e.g., students and employees); examples include social support, school-based physical education, community-wide information, and motivational campaigns, access to places for PA, environmental opportunities, etc.

## 5. Conclusions

The present study confirms previous data on the negative impact of the SARS-CoV-2 lockdown on the levels of PA and sedentary behavior among adults living in Italy. It also provided new evidence on the extent of PA reduction and on the increase in sedentary behavior across different subgroups. This approach allowed to identify individuals most in need of preventive PA interventions. These findings should be extended and applied to other future social emergencies or crises or to individual health conditions that require people to stay at home for prolonged periods of time.

## Figures and Tables

**Table 1:** Characteristics of the sample (*N* = 2596).

Variable	Median (IQR)	*n* (%)
	[Mean ± SD]	
Age, years	24 (11)	-
	[30 ± 12]	
Gender	-	
Women		1837 (70.8)
Men		759 (29.2)
Role	-	
Employees		621 (23.9)
Students		1975 (76.1)
SARS-CoV-2 infection, *n* of Yes	-	44 (1.7)
BMI, kg/m^2 1^	21.9 (4.1)	-
	[22.4 ± 3.7]	
BMI, categories ^1^	-	
Underweight		217 (8.6)
Normal weight		1854 (73.4)
Pre-obesity/Obesity		454 (18.0)
Level of education	-	
Middle school		6 (0.2)
High school diploma		1414 (54.5)
University bachelor’s degree		575 (22.1)
University master’s degree		264 (10.2)
Postgraduate education (Ph.D., specialization schools, etc.)		337 (13.0)
Sports, *n* of Yes	-	1842 (71.0)
Sport characteristics	-	
Individual sports		1464 (78.3)
Sports teams		405 (21.7)

^1^ Data were based on *n* = 2525.

**Table 2:** Comparison of categorical IPAQ scores before and during the lockdown.

		During Lockdown			
	Level of PA	Physically Inactive	Active	Highly Active	Total
Before lockdown	Physically inactive	186 (65.7)	57 (20.1)	40 (14.1)	283 (10.9)
	Active	508 (40.3)	559 (44.3)	194 (15.4)	1261 (48.6)
	Highly active	216 (20.5)	315 (29.9)	521 (49.5)	1052 (40.5)
	Total	910 (35.0)	931 (35.9)	755 (29.1)	

Data are reported as *n* (%); *p* value < 0.001 by Stuart-Maxwell test for marginal homogeneity. Elements below the diagonal represent individuals who engaged in less PA; elements above the diagonal represent individuals who engaged in more PA.

**Table 3:** Comparison of continuous IPAQ values before and during the lockdown (total PA, subtypes of PA, and sedentary behavior).

		Before Lockdown	During Lockdown	Δ	Difference within Groups	Difference between Groups
Group analysis		Total PA (MET-minutes/week)				
Total	-	2307 (1322–3804)	1367 (500–2619)	713 (0–1919)	Z= −25.42 **; ES = 0.50	-
Role	Students	2424 (1386–4158)	1473 (600–2838)	711 (0–2004)	Z = −21.54 **; ES = 0.48	Z = −0.32
	Employees	1884 (1159–2994)	924 (364–1873)	718 (0–1638)	Z = −13.75 **; ES = 0.55	
Gender	Women	2106 (1173–3444)	1260 (480–2466)	643 (−80–1732)	Z = −19.54 **; ES = 0.46	Z = −5.00 **; ES = 0.10
	Men	2895 (1724–4692)	1600 (558–3033)	933 (0–2310)	Z = −16.42 **; ES = 0.60	
BMI categories ^1^	Underweight (*n* = 217)	2106 (1045–3465)	1196 (398–2352)	561 (0–1840)	Z = −6.63 **; ES = 0.45	H = 7.62 *; η^2^ = 0.002
	Normal-weight (*n* = 1854)	2394 (1390–4026)	1440 (600–2699)	759 (0–1983)	Z = −22.49 **; ES = 0.52	
	Pre-obese/Obesity (*n* = 454)	2043 (1040–3170)	1070 (360–2358)	632 (−130–1642)	Z = −9.05 **; ES = 0.42	
Play sport	Yes	2826 (1794–4429)	1530 (720–2880)	977 (0–2280)	Z = −25.48 **; ES = 0.59	Z = −12.53 **; ES = 0.25
	No	1282 (676–2131)	840 (231–1922)	185 (−278–1004)	Z = −6.12 **; ES = 0.22	
Group analysis		Sedentary behavior ^2^ (minutes/day)				
Total	-	349 (240–480)	440 (300–600)	−60 (−180–0)	Z = −27.41 **; ES = 0.57	-
Role	Students	300 (180–480)	420 (300–557)	−71 (−180–0)	Z = −23.77 **; ES = 0.58	Z = −1.55
	Employees	360 (300–490)	480 (360–600)	−60 (−150–0)	Z = −13.67 **; ES = 0.57	
Gender	Women	350 (240–480)	440 (300–600)	−60 (−180–0)	Z = −21.91 **; ES = 0.55	Z = −0.84
	Men	300 (240–480)	440 (300–600)	−80 (−180–0)	Z = −16.65 **; ES = 0.63	
BMI categories ^1^	Underweight (*n* = 178)	334 (240–480)	440 (300–600)	−60 (−180–0)	Z = −7.95 **; ES = 0.60	H = 3.43
	Normal-weight (*n* = 1636)	349 (240–480)	440 (300–600)	−80 (−180–0)	Z = −23.52 **; ES = 0.58	
	Pre-obese/Obesity (*n* = 400)	350 (240–480)	480 (300–600)	−60 (−175–0)	Z = −11.16 **; ES = 0.56	
Play sport	Yes	330 (200–480)	440 (300–600)	−80 (−180–0)	Z = −24.43 **; ES = 0.60	Z = −3.07 *; ES = 0.06
	No	360 (240–480)	440 (300–600)	−60 (−180–0)	Z = −12.67 **; ES = 0.50	
Group analysis		Vigorous PA (MET-minutes/week)				
Total	-	960 (0–1920)	480 (0–1680)	0 (−120–830)	Z = −10.01 **; ES = 0.20	-
Role	Students	960 (0–2160)	720 (0–1920)	0 (−240–960)	Z = −8.14 **; ES = 0.18	Z = −0.27
	Employees	480 (0–1440)	80 (0–960)	0 (0–580)	Z = −6.56 **; ES = 0.26	
Gender	Women	720 (0–1920)	480 (0–1440)	0 (−240–640)	Z = −5.42 **; ES = 0.13	Z = −5.96 **; ES = 0.12
	Men	1440 (480–2880)	800 (0–2160)	0 (0–1280)	Z = −9.56 **; ES = 0.35	
BMI categories ^1^	Underweight	480 (0–1740)	240 (1–1440)	0 (0–480)	Z = −2.18 *; ES = 0.15	H = 1.29
	Normal-weight	960 (0–2160)	720 (0–1800)	0 (−160–960)	Z = −9.08 **; ES = 0.21	
	Pre-obese/Obesity	800 (0–1920)	240 (0–1440)	0 (0–720)	Z = −3.62 **; ES = 0.17	
Play sport	Yes	1440 (480–2400)	800 (0–1920)	160 (0–1200)	Z = −14.73 **: ES = 0.34	Z = −14.50 **; ES = 0.28
	No	0 (0–320)	0 (0–960)	0 (−480–0)	Z = −7.84 **; ES = 0.29	
Group analysis		Moderate PA (MET-minutes/week)				
Total	-	240 (0–720)	240 (0–640)	0 (−240–240)	Z = −1.32	-
Role	Students	240 (0–720)	240 (0–720)	0 (−240–240)	Z = −6.66	Z = −1.79
	Employees	240 (0–600)	240 (0–510)	0 (−120–240)	Z = −1.62	
Gender	Women	240 (0–640)	240 (0–720)	0 (−240–200)	Z = −1.34	Z = −4.19 **; ES = 0.08
	Men	240 (0–720)	240 (0–560)	0 (−120–280)	Z = −4.50 **; ES = 0.16	
BMI categories ^1^	Underweight	240 (0–680)	240 (0–720)	0 (−140–120)	Z = −0.56	H = 2.91
	Normal-weight	240 (0–720)	240 (0–720)	0 (−240–240)	Z = −1.94 *; ES = 0.04	
	Pre-obese/Obesity	160 (0–540)	190 (0–560)	0 (−240–160)	Z = −0.56	
Play sport	Yes	360 (0–720)	240 (0–720)	0 (−160–280)	Z = −5.92 **; ES = 0.14	Z = −8.71 **; ES = 0.17
	No	0 (0–360)	160 (0–560)	0 (−320–0)	Z = −7.64 **; ES = 0.28	
Group analysis		Walking (MET-minutes/week)				
Total	-	693 (396–1386)	99 (0–396)	495 (66–990)	Z = −35.29 **; ES = 0.69	-
Role	Students	693 (396–1386)	66 (0–396)	495 (66–1023)	Z = −30.49 **; ES = 0.69	Z = −0.85
	Workers	693 (396–1386)	99 (0–396)	462 (99–990)	Z = −17.81 **; ES = 0.71	
Gender	Women	693 (396–1386)	66 (0–396)	495 (99–1040)	Z = −30.03 **; ES = 0.70	Z = −2.47 *; ES = 0.05
	Men	693 (347–1386)	99 (0–462)	429 (0–924)	Z = −18.52 **; ES = 0.67	
BMI categories ^1^	Underweight	792 (346–1386)	99 (0–396)	495 (41–990)	Z = −10.51 **; ES = 0.71	H = 10.81 *; η^2^ = 0.004
	Normal-weight	693 (396–1386)	99 (0–396)	495 (99–1040)	Z = −30.31 **; ES = 0.70	
	Pre-obese/Obesity	693 (330–1188)	99 (0–445)	396 (0–924)	Z = −13.34 **; ES = 0.63	
Play sport	Yes	693 (396–1386)	99 (0–396)	462 (99–990)	Z = −29.89 **; ES = 0.70	Z = −0.57
	No	792 (396–1386)	66 (0–396)	495 (0–1064)	Z = −18.74 **; ES = 0.68	

Data are presented as Median (Q1–Q3); Δ is the difference between before and during the lockdown. ^1^ data on *n* = 2525; ^2^ data on *n* = 2275; ** *p* < 0.001; * *p* ≤ 0.05.

## Data Availability

The data presented in this study are available on request from the corresponding author.
